# Associations between mental health problems and risky oral and sexual behaviour in adolescents in a sub-urban community in Southwest Nigeria

**DOI:** 10.1186/s12903-021-01768-w

**Published:** 2021-08-16

**Authors:** Morenike Oluwatoyin Folayan, Olaniyi Arowolo, Boladale Mapayi, Nneka Maureen Chukwumah, Michael A. Alade, Randa H. Yassin, Maha El Tantawi

**Affiliations:** 1 Department of Child Dental Health, Obafemi Awolowo University, Ile-Ife, Nigeria; 2 Obafemi Awolowo University Teaching Hospitals Complex, Ile-Ife, Nigeria; 3 Department of Mental Health, Obafemi Awolowo University, Ile-Ife, Nigeria; 4 Department of Preventive Dentistry, School of Dentistry, University of Benin, Benin City, Nigeria; 5 Department of Pediatric Dentistry and Dental Public Health, Faculty of Dentistry, Alexandria University, Alexandria, Egypt

**Keywords:** Oral health, Mental health, Sexual health, Adolescents, Nigeria

## Abstract

**Background:**

This study determined the association between mental health and risky oral health and sexual health behaviours.

**Methods:**

A household cross-sectional survey was conducted in Ile-Ife, Nigeria between December 2019 and January 2020. Data were collected from 10 to 19-year-old on the sociodemographic profile (age, sex at birth and socioeconomic status); mental health problems (psychological distress, depressive symptoms and suicidal ideation); and mental (smoking habit, consumption of alcohol, use of psychoactive substances), sexual (history of vaginal or anal sexual intercourse; transactional sex, multiple sex partners, use of condom at last sexual intercourse) and oral (frequency of daily tooth brushing, daily frequency of consumption of refined carbohydrate in-between-meals, frequency of use of dental floss, history of dental service utilization in the last 12 months and dental anxiety) health risk factors. Binary logistic regression analysis was conducted to determine the association between risky oral (neglecting to brush twice daily and frequent consumption of refined carbohydrates in-between-meals), and sexual (neglecting to use condoms during the last sex act and having multiple sex partners) health behaviours as outcome variables, and mental health status as the explanatory variables. An ordinal logistic regression model was also developed where the outcome variable was the number of risky health behaviours. The models were adjusted for the socio-demographic variables and history of dental service utilisation in the last 12 months of the survey.

**Results:**

High psychological distress was significantly associated with lower odds of frequent consumption of refined carbohydrates in-between-meals (AOR = 0.32; 95%CI 0.23, 0.47), and having multiple sex partners (AOR = 0.10; 95%CI 0.02, 0.57); but higher odds of having a higher number of risky behaviours (AOR = 3.04; 95%CI 2.13, 4.33). Having depressive symptoms was significantly associated with higher odds of neglecting to use condom at the last sexual intercourse (AOR = 7.20; 95%CI 1.94, 26.76) and having multiple partners (AOR = 95.43; 95%CI 24.55, 370.90). Suicidal ideation was significantly associated with lower odds of neglecting to use condom at the last sexual intercourse (AOR = 0.00; 95%CI 0.00, 0.00) and having multiple sex partners (AOR = 0.00; 95%CI 0.00, 0.00).

**Conclusion:**

The associations between psychological distress and oral and sexual health risk behaviours in adolescents seem complex and need to be studied further.

## Background

About 10–25% of adolescents experience mental disorders worldwide [1]. Mental health disorders are disorders that affect the cognitive, behavioural, and emotional well-being of individuals. These include psychological distress, depressive symptoms and suicidal ideation [1]. Half of all mental illnesses begin by 14 years of age [2]. If untreated, mental health conditions severely influence children’s development, their educational attainments and their potential to live fulfilling and productive lives. Mental health problems are increasing substantially, particularly among girls and young women and so is self-harm presentations in 13–16-year-old girls [3].

The mental health status of adolescents’ can affect the development of their capacity for sexual intimacy, sexual identity and reproduction potential and vice versa [4]. Adolescents who had been physically and sexually abused have increased suicidal tendencies [5], and women affected by physical and sexual abuse also have poorer general health and higher prevalence of sexually transmitted infection [6, 7]. Poor sexual health also affects the individual’s mental health with increased risk of low self-esteem, depression and suicidal tendencies [8].

The prevalence of suicidal ideation is quite high in adolescents [9]—about 20–30% of adolescents contemplate suicide—and a risk factor for completing suicide [10, 11]. A history of sexual abuse is an independent risk factor for suicidal ideation in adolescents [12]. Depression is also a linking factor between suicidal ideation and poor sexual health [12].

Adolescents’ mental health also affects their oral health and vice-versa [13]. Good occlusion boosts the self-esteem and social lives of adolescents [14] and improves the quality of life and ability to handle stressful events [15]. Depressed adolescents are less likely to care about their oral health. The prevalence of poor oral health is high in adolescence [16] with associated risk of negative impact on general health and wellbeing in adulthood if not managed [17].

Folayan et al. [13] had postulated a link between the oral, mental, sexual and reproductive health of adolescents and proposed that managing these three disease entities may be an appropriate common risk integrated approach for health care for adolescents. Integrated health care is a cost-effective approach that can promote access to quality health care throughout life course. This study aims to determine empirically, if mental health is associated with risky oral health and sexual health behaviours.

## Methods

### Study population and study design

This was a secondary analysis of a dataset collected to determine the risk factors for caries in adolescents. The data were collected through a household cross-sectional survey conducted between December 2018 and January 2019 in Ife Central Local Government Area of Osun, State Ile-Ife, a semi-urban community in Nigeria. Adolescents aged 10–19-years from whom parental consents/ assents/ individual informed consents were obtained where appropriate, were eligible to participate in the study. Adolescents who were critically ill and could not give independent responses to the study survey were excluded from participation. Recruitment of participants continued until the sample size for the study was reached [18, 19].

### Sample size and sampling technique

The minimal sample size for the study was calculated with the formula proposed by Araoye [20]. With a caries prevalence of 13.9% [21] a margin of error of 5%, and a confidence level of 95%, the minimum sample size was 1323 adolescents. Adolescents were recruited with a multi-stage sampling technique. First, 70 of the 700 enumeration areas in Ife Central Local Government Area were sampled with the simple random technique. Next, every other household in the selected enumeration areas was identified as an eligible household. Finally, in each household, one adolescent who met the inclusion criteria was recruited for study participation. Whenever a household declined to participate, the next eligible household was substituted.

### Study procedure

An interviewer administered questionnaire was used for the data collection process. The questionnaire was composed of tools validated for use in Nigeria to measure oral, mental, sexual and reproductive health. The questionnaire was piloted with 10 adolescents for timing and clarity after being tested with four dentists for content validation. The questionnaire took an average of 22 min to fill. Participants were administered the questionnaire after the consent was explained and the consent form signed. Questionnaire was administered in English. Participants were also free to skip questions they were not willing to answer. At the end of the interview, participants were given a sachet of toothpaste which costs about $0.24.

### Demographics

Information on age, sex at birth and socioeconomic status was collected. The socioeconomic status of each respondent was determined using the index developed by Olusanya et al. [22], which had been used in a previous study in the study environment [23]. The index is based on multiple items combining the mother’s level of education with the father’s educational level and occupation. For this study, data were collected on the educational levels and professions of respondents’ parents. The study participants’ mother’s level of education was classified as follows: no formal education, Quranic and primary school education (score 2); secondary school education (score 1) and tertiary education (scored 0). The father’s occupation was also categorized into three levels: civil servants or skilled professionals with a tertiary level of education (score 1); civil servants or skilled professionals with a secondary level of education (score 2); unskilled, unemployed, students, and civil servants or skilled professionals with a primary and or Quranic education (scored 3). The social class of the adolescent was determined by adding the score of the mother’s level of education to that of the father’s occupation. Each adolescent was allocated into social classes I–V (class I, upper class; class II, upper middle class; class III, middle class; class IV, lower middle class; class V, lower class). For analysis, the categories were collapsed into: class I: high socioeconomic level, class 2: middle socioeconomic level and class 3: low socioeconomic level. When an adolescent had lost a parent, their socioeconomic status was determined using the status of the living parent.

### Mental health

The mental health of respondents was assessed to determine if respondents had mental health problems and the severity of the problems. The presence of psychological distress, depressive symptoms and suicidal ideation were assessed.

#### Psychological distress

Study participants’ psychological distress status was assessed using the General Health questionnaire (GHQ). The tool has been validated for use in Nigeria [24, 25]. The 12-item version is useful in settings where, because of widespread illiteracy, the questionnaire has to be read to respondents. The GHQ is scored using the Likert method (0–1–2–3). Each item is accompanied by four possible responses, ‘not at all’, ‘no more than usual’, ‘rather more than usual’ and ‘much more than usual’. The total possible score on the GHQ-12 ranges from 0 to 36 with cut-off point of greater or equal to 7/8 in Nigeria. Participants with score > 7 were classified as having high psychological distress and those with score ≤ 7 were classified as having low psychological distress. In a study by Gureje [26], the alpha coefficient of the GHQ-12 was 0.82.

#### Depressive symptoms

A nine-item questionnaire, the Patient Health Questionnaire, assessed depression using the DSM-IV criteria. Scores for each of the nine-item ranged from “0” (not at all) to “3” (nearly every day) with total scores ranging from 0 to 27. Total scores 0–4 indicated no depression, scores 5 and higher indicated the presence of depression of any degree [27]. It has good concurrent validity with Beck’s depression inventory (0.61), and a one-month test–retest reliability of 0.89 among young Nigerian adults [28].

#### Suicidal ideation

Data on suicidal ideation and attempt was obtained using the Suicidal Behaviour Questionnaire Revised. It consists of four items and has been validated in a high school sample with a Cronbach’s alpha of 0.88 [29]. We used the first question “have you ever thought about killing yourself” for analysis recoded as ‘yes’ (Yes, just a brief passing thought; Yes, had a plan but did not do it; Yes, had a plan and really wanted to do it) and ‘no’ (never).

### Mental health risk factors

#### Smoking habit

The questionnaire requested information on the respondents’ habits of cigarette smoking. The questions had six alternatives—No, never; No, I used to, but I quit; Yes, once a month or less; Yes, a few times (2–3) a month; Yes, a few times (2–3) a week; Yes, once a day or more. All those who chose options ‘Yes, once a month or less; Yes, a few times (2–3) a month; Yes, a few times (2–3) a week; Yes, once a day or more’ were classified as smokers [30] and those who chose ‘No, never and No, I used to’ were classified as non- smokers.

#### Consumption of alcohol

Respondents were asked about their frequency of alcohol intake (Every day, once a week, less than once a week, never, not sure and no response). The responses were categorized into yes (Every day, once a week, less than once a week) and no (never, not sure).

#### Use of psychoactive substances

Information on the use of psychoactive substances (marijuana, solvent glue, cocaine, heroin, tramadol, codeine, injecting cocaine or heroin using a syringe and needle) was collected. The responses were dichotomized into ‘No’ when there was no indication of psychoactive substance use, and ‘Yes’ if there was indication of using any of these drugs.

### Sexual health risk factors

Participants were asked if they had ever had vaginal or anal sexual intercourse; had sex in exchanged for money, a place to stay, or material goods (transactional sex); number of male and female sex partners and if a condom was used at the last vaginal and/or anal intercourse. These measures were similar to others that have been found reliable and valid in previous research [31]. Information on unprotected oral sexual intercourse was not collected as oral sex is not considered a behaviour that increases the risk for becoming pregnant or making a partner pregnant though it is a route for contracting a sexually transmitted infection [32]. The risk of contracting most sexually transmitted infection from oral sex is lower than for vaginal or anal sex [33].

### Oral health risk factors

Information was collected on frequency of daily tooth brushing, daily frequency of consumption of refined carbohydrate in-between-meals, frequency of use of dental floss, history of dental service utilization in the last 12 months and dental anxiety.

#### Tooth brushing

Respondents were asked to indicate the frequency of tooth brushing using the following alternatives—irregularly or never, once a week, a few (2–3) times a week, once a day, and more than once a day. Respondents who chose the options ‘irregularly or never, once a week, a few (2–3) times a week, once a day’ were classified as not having undertaken preventive dental care [30].

#### Consumption of refined carbohydrate in-between-meals

Respondents were also asked to indicate the frequency of consuming sugar-containing snacks or drinks between main meals using the following alternatives—About 3 times a day or more, about twice a day, about once a day, occasionally, not every day, rarely or never eat between meals. Respondents who chose the options ‘About 3 times a day or more, about twice a day, about once a day’, were classified as not having undertaken preventive dental care [30].

#### Use of dental floss

Respondents were also asked to indicate how often dental floss was used to clean their teeth using the following alternatives—Not at all, occasionally, a few (2–3) times a week, once in a day, more than one time in a day. Respondents, who chose the options ‘Not at all, occasionally, a few (2–3) times a week’, were classified as not having undertaken preventive dental care [30].

#### Dental service utilization

Respondents were also asked to indicate the time of the last check-up using the following alternatives—within the last 6 months, more than 6 months to 1 year ago, more than 1–2 years ago, more than 2–5 years ago, more than 5 years, never, do not remember. Attending a dental check-up within the last 12 months was defined as preventive care use. Respondents who chose the options ‘more than 1–2 years ago, more than 2–5 years ago, more than 5 years, never, do not remember’ were classified as not having undertaken preventive dental care [30].

#### Dental anxiety

Dental anxiety refers to patients' specific response towards dental situation-associated stress [34]. It was measured using the Dental Anxiety Scale a reliable, valid questionnaire used to assess dental anxiety [35]. The scale has four questions with five-point Likert scale responses. The level of anxiety was assessed as a summation of the scores of all questions. Possible scores range from 4 to a maximum of 20. The scores were coded as follows, 4–8 as low anxiety, 9–12 as moderate anxiety, 13–14 as high anxiety, and 15–20 as severe anxiety bordering on phobia. The scale had been validated for use in Nigeria [36]. The Cronbach’s alpha for this study was 0.92.

### Data analysis

Descriptive analysis was conducted to determine the proportion of adolescents with each sociodemographic variable (age, sex, socioeconomic status,), mental health problems (psychological distress, depression, suicidal ideation), and mental (smoking, consumption of alcohol, intake of psychoactive substances) sexual (being sexually active, transactional sex, number of sex partners, use of condom at last sexual intercourse) and oral (dental anxiety, tooth brushing frequency, frequency of flossing, intake of refined carbohydrate in-between-meals, dental service utilization in the last 12 months) health risk factors. Bivariate analysis was conducted to determine the association between mental health problems, sexual and oral health risk behaviours and sociodemographic factors.

Binary logistic regression models were developed to assess the relationship between the risky oral health (neglecting to brush twice daily and frequent consumption of refined carbohydrates in-between-meals), and sexual health (neglecting to use condoms during the last sex act and having multiple sex partners) behaviours as outcome variables; and the explanatory variables (psychological distress, depressive symptoms and suicidal ideation). An ordinal logistic regression model was also developed where the outcome variable was the number of risky behaviours. The number of risky health behaviours was computed by counting how many of four risky behaviours (2 risky oral health behaviours namely: neglecting to brush teeth twice daily and consuming refined carbohydrates in between meals and 2 risky sexual health behaviours namely: neglecting to use condoms at the last sex act and having multiple sex partners) and individual self-identified to have. The possible scores for risky health behaviours ranged from 0 to 4. The models were adjusted for confounders (sex, age and socioeconomic status) and visit to the dentist in the last 12 months before the survey. The estimated coefficients, expressed as adjusted odds ratios (AOR) and their 95% confidence intervals (CIs), were calculated. The statistical analyses were conducted using IBM SPSS for Windows version 22.0 (IBM Corp., Armonk, N.Y., USA). Statistical significance was inferred at *p* ≤ 0.05.

#### Ethical consideration

Ethical approval for the study was obtained from the ethics and research committee of the Institute of Public Health, Obafemi Awolowo University, Ile-Ife, Nigeria (IPHOAU/12/669). Approval for conduct of the study was obtained from the Local Government Authority prior to commencement of the study. All methods were carried out in accordance with relevant guidelines and regulations. Informed consent was obtained from the parent of each study participant age 10–11 years old prior to enrolment. Parental consent and participant assent were obtained for those 12–13-years-old. Consent was obtained from study participants 14–19 years in line with guidance from the Federal Ministry of health [37]. Efforts were made to minimize the risks of loss of confidentiality by ensuring that anonymized data collection was done privately and was collected with an electronic data platform. Study participants discomfort with the personal nature of questions was limited by ensuring field workers were trained on how to ask sensitive questions and clarify non-verbal cues observed during the interviews.

## Results

Although there were 1472 respondents, complete data for all the variables of interest were available for 964 adolescents with a mean age of 14.6 years. Table 1 highlights the sociodemographic, oral, mental and sexual health profile of respondents. The majority (56.1%) of respondents were males. There were 315 (32.7%) respondents with high socioeconomic status, 876 (90.9%), adolescents who did not brush twice a day, 606 (62.9%) who consumed refined carbohydrates in-between-meals daily, 849 (88.1%) who did not use dental floss, 953 (89.9%) who did not visit the dentist in the last 12 months of the study and 194 (20.1%) who had severe dental anxiety. Also, 76 (7.9%) respondents were sexually active, and of these, 5 (6.6%) had a history of transactional sex, 25 (32.9%) did not use a condom at the last sexual intercourse and 17 (22.4%) had multiple sex partners. In addition, there were 197 (20.4%) respondents with high psychological distress, 30 (3.1%) who had suicidal ideations, 63 (6.5%) with depressive symptoms, 92 (9.5%) who consumed alcohol, 43 (4.5%) who used psychoactive drugs and 10 (1.0%) who smoked cigarettes.

**Table 1:** Sociodemographic, oral, mental and sexual health profile of adolescents 10–19 years old resident in Ile-Ife, Nigeria [N = 964]

Factors	n (%)
*Sociodemographic factors*	
Age	
Mean (SD)	14.6 (2.7)
Sex	
Male	541 (56.1)
Female	423 (43.9)
Socioeconomic status	
High	315 (32.7)
Middle	329 (34.1)
Low	320 (33.2)
*Oral health risk factors*	
Brushing twice a day	
Yes	88 (9.1)
No	876 (90.9)
Daily consumption of refined carbohydrates in-between-meals	
Yes	606 (62.9)
No	358 (37.1)
Use of dental floss	
Yes	115 (11.9)
No	849 (88.1)
Visited the dentist in the last year	
Yes	11 (1.1)
No	953 (89.9)
Dental anxiety	
Low	293 (30.4)
Moderate	367 (38.1)
High	110 (11.4)
Severe	194 (20.1)
*Sexual health risk factors*	
Sexually active	
Yes	76 (7.9)
No	888 (92.1)
Transactional sex (n = 76)	
Yes	5 (6.6)
No	71 (93.4)
Did not use condom at last sexual intercourse (n = 76)	
Yes	25 (32.9)
No	51 (67.1)
Multiple sex partners (n = 76)	
Yes	17 (22.4)
No	59 (77.6)
*Mental health*	
Psychological distress	
High	197 (20.4)
Low	767 (79.6)
Suicidal ideation	
Yes	30 (3.1)
No	934 (96.9)
Depressive symptoms	
Yes	63 (6.5)
No	901 (93.5)
*Mental health risk factors*	
Consumption of alcohol	
Yes	92 (9.5)
No	872 (90.5)
Use of psychoactive substances	
Yes	43 (4.5)
No	921 (95.5)
Cigarette smoking	
Yes	10 (1.0)
No	954 (99.0)

Table 2 shows that there were significantly more adolescents with high psychological distress than those with low psychological distress who used dental floss (21.3% vs. 9.5%; *p* < 0.001), had severe dental anxiety (25.9% vs. 18.6%; *p* < 0.001), were sexually active (12.2% vs. 6.8%; *p* = 0.01), consumed alcohol (15.7% vs. 8.0%; *p* = 0.001), and smoked cigarettes (2.5% vs. 0.7%; *p* = 0.04). Also, there were significantly more adolescents with high socioeconomic status who had low than high psychological distress (25.4% and 34.6%; *p* = 0.04) and consume refined carbohydrates in-between-meals daily (43.1% and 67.9%; *p* < 0.001); and more of those who were older than young that had high psychological distress (15.5 years vs. 14.4 years; *p* < 0.001).

**Table 2:** Sociodemographic, oral, sexual and mental health risk factors associated with mental health problems in adolescents 10–19 years old resident in Ile-Ife, Nigeria [N = 964]

Risk factors	Psychological distress			Depressive symptoms			Suicidal ideation		
	High n (%)	Low n (%)	*P* value	Present n (%)	Absent n (%)	*P* value	Present n (%)	Absent n (%)	*P* value
*Sociodemographic factors*									
Age									
Mean (SD)	15.5 (2.5)	14.4 (2.6)	< 0.001*	16.0 (2.4)	14.5 (2.6)	< 0.001*	16.0 (2.33)	14.6 (2.7)	0.003*
Sex									
Male	102 (51.8)	439 (57.2)	0.17	28 (44.4)	513 (56.9)	0.05	17 (56.7)	524 (56.1)	0.95
Female	95 (48.2)	328 (42.8)		35 (55.6)	388 (43.1)		13 (43.3)	410 (43.9)	
Socioeconomic status									
High	50 (25.4)	265 (34.6)	0.04*	25 (39.7)	290 (32.2)	0.32	13 (43.3)	302 (32.3)	0.25
Middle	78 (39.6)	251 (32.7)		22 (34.9)	307 (34.1)		11 (36.7)	318 (34.0)	
Low	69 (35.0)	251 (32.7)		16 (25.4)	304 (33.7)		6 (20.0)	314 (33.6)	
*Oral health risk factors*									
Brushing twice a day									
Yes	23 (11.6)	65 (8.5)	0.16	10 (15.9)	78 (8.7)	0.06	3 (10.0)	85 (9.1)	0.75
No	174 (88.3)	702 (91.5)		53 (84.1)	823 (91.3)		27 (90.0)	849 (90.9)	
Daily consumption of refined carbohydrate in-between-meals									
Yes	85 (43.1)	521 (67.9)	< 0.001*	32 (50.8)	574 (63.7)	0.04*	19 (63.3)	587 (62.8)	0.96
No	112 (56.9)	246 (32.1)		31 (49.2)	327 (36.3)		11 (36.7)	347 (37.2)	
Use of dental floss									
Yes	42 (21.3)	73 (9.5)	< 0.001*	8 (12.7)	107 (11.9)	0.84	3 (10.0)	112 (12.0)	1.00
No	155 (78.7)	694 (90.5)		55 (87.3)	794 (88.1)		27 (90.0)	822 (88.0)	
Dental service utilization									
Yes	2 (1.0)	9 (1.2)	1.00	1 (1.6)	10 (1.1)	0.53	0 (0.0)	11 (1.2)	1.00
No	195 (99.0)	758 (98.8)		62 (98.4)	891 (98.9)		30 (100.0)	923 (98.8)	
Dental anxiety									
Low	49 (24.9)	244 (31.8)	< 0.001*	21 (33.3)	272 (30.2)	0.24	8 (26.7)	285 (30.5)	0.94
Moderate	62 (31.5)	305 (39.8)		23 (36.5)	344 (38.2)		13 (43.3)	354 (37.9)	
High	35 (17.8)	75 (9.8)		11 (17.5)	99 (11.0)		3 (10.0)	107 (11.5)	
Severe	51 (25.9)	143 (18.6)		8 (12.7)	186 (20.6)		6 (20.0)	188 (20.1)	
*Sexual health risk factors*									
Sexually active									
Yes	24 (12.2)	52 (6.8)	0.01*	19 (30.2)	57 (6.3)	< 0.001*	3 (100.0)	73 (7.8)	0.73
No	173 (87.8)	715 (93.2)		44 (69.8)	844 (93.7)		27 (90.0)	861 (92.2)	
Transactional sex (n = 76)									
Yes	3 (12.5)	2 (3.8)	0.32	3 (15.8)	2 (3.5)	0.10	0 (0.0)	5 (6.8)	1.00
No	21 (87.5)	50 (96.2)		16 (84.2)	55 (96.5)		3 (100.0)	68 (93.2)	
Did not use condom at last sexual intercourse (n = 76)									
Yes	6 (25.0)	19 (36.5)	0.32	6 (31.6)	19 (33.3)	0.89	0 (0.0)	25 (34.2)	0.55
No	18 (75.0)	33 (63.5)		13 (68.4)	38 (66.7)		3 (100.0)	48 (65.8)	
Multiple sex partners (n = 76)									
Yes	6 (25.0)	11 (21.2)	0.71	8 (42.1)	9 (15.8)	0.02*	0 (0.0)	17 (23.3)	1.00
No	18 (75.0)	41 (78.8)		11 (57.9)	48 (84.2)		3 (100.0)	56 (76.7)	
*Mental health risk factors*									
Consumption of alcohol									
Yes	31 (15.7)	61 (8.0)	0.001*	18 (28.6)	74 (8.2)	< 0.001*	10 (33.3%)	82 (8.8%)	< 0.001*
No	166 (84.3)	706 (92.0)		45 (71.4)	827 (91.8)		20 (66.7%)	852 (91.2%)	
Use of psychoactive substances									
Yes	13 (6.6)	30 (3.9)	0.10	12 (19.0)	31 (3.4)	< 0.001*	4 (13.3%)	39 (4.2%)	0.04*
No	184 (93.4)	737 (96.1)		51 (81.0)	870 (96.6)		26 (86.7%)	895 (95.8%)	
Cigarette smoking									
Yes	5 (2.5)	5 (0.7)	0.04*	5 (7.9)	5 (0.6)	< 0.001*	2 (6.7)	8 (0.9)	0.04*
No	192 (97.5)	762 (99.3)		58 (92.1)	896 (99.4)		28 (93.3)	926 (99.1)	

^*^Statistically significant at *p* < 0.05

Also, there were significantly more respondents with depressive symptoms than those without depressive symptom who were older (16.0 years vs. 14.5 years; *p* < 0.001), more likely to be sexually active (30.2% vs. 6.3%; *p* < 0.001), have multiple sex partners (42.1% vs. 15.8%; *p* = 0.02), consumed alcohol (28.6% vs. 8.2%; *p* < 0.001), use psychoactive substances (19.0% vs. 3.4%; *p* < 0.001) and smoked cigarettes (7.9% vs. 0.6%; *p* < 0.001). However, adolescents with depressive symptoms were less likely to consume refined carbohydrates in-between-meals daily (50.8% vs. 63.7%; *p* = 0.04).

In addition, there were more respondents who had suicidal ideation than those who did not have suicidal ideation who were older (16.0 years-old vs. 14.6 years-old; *p* = 0.003), consumed alcohol (33.3% vs. 8.8%; *p* < 0.001), use psychoactive drugs (13.3% vs. 4.2%; *p* = 0.04) and smoked cigarettes (6.7% vs. 0.9%; *p* = 0.04).

Table 3 shows that high psychological distress was significantly associated with lower odds of daily consumption of refined carbohydrates in-between-meals (AOR: 0.32; 95%CI 0.23, 0.47), and having multiple sex partners (AOR: 0.10; 95%CI 0.02, 0.57); but higher odds of having a higher number of risky behaviours (AOR: 3.04; 95%CI 2.13, 4.33). Having depressive symptoms was significantly associated with higher odds of not using condom at the last sexual intercourse (AOR: 7.20; 95%CI 1.94, 26.76) and having multiple partners (AOR: 95.43; 95%CI 24.55, 370.90). Suicidal ideation was significantly associated with lower odds of not using condom at the last sexual intercourse (AOR = 0.00, 95%CI 0.00, 0.00) and having multiple sex partners (AOR: 0.00; 95%CI 0.00, 0.00).

**Table 3:** Association between mental health problems and oral and sexual risky behaviours (n = 964)

Variables	AOR (95% CI)				
	Neglecting tooth brushing	Frequent consumption of refined carbohydrates	Did not use condom at last sexual intercourse	Having multiple sex partners	Higher number of risky behaviours
Gender					
Males	1.64 (1.04, 2.59)*	0.94 (0.71, 1.24)	0.74 (0.33, 1.67)	2.23 (0.57, 8.68)	0.93 (0.72, 1.20)
Females	1.00	1.00	1.00	1.00	1.00
Age	1.05 (0.96, 1.15)	0.98 (0.93, 1.03)	1.68 (1.35, 2.10)*	2.83 (2.08, 3.85)*	0.96 (0.92, 1.01)
Socioeconomic status					
High	0.68 (0.38, 1.24)	0.34 (0.24, 0.49)*	1.47 (0.47, 4.62)	0.57 (0.12, 2.72)	2.61 (1.87, 3.64)*
Medium	0.69 (0.39, 1.21)	0.50 (0.35, 0.70)*	1.47 (0.47, 4.59)	2.27 (0.53, 9.81)	1.84 (1.35, 2.51)*
Low	1.00	1.00	1.00	1.00	1.00
Dental visits in the past year					
Yes	0.09 (0.02, 0.30)*	1.93 (0.49, 7.52)	0.00 (0.000, 0.00)*	0.00 (0.000, 0.00)*	2.99 (0.61, 14.72)
No	1.00	1.00	1.00	1.00	1.00
Psychological distress					
High	0.73 (0.41, 1.29)	0.32 (0.23, 0.47)*	0.36 (0.09, 1.46)	0.10 (0.02, 0.57)*	3.04 (2.13, 4.33)*
Low	1.00	1.00	1.00	1.00	1.00
Depressive symptoms					
Yes	0.58 (0.24, 1.36)	0.99 (0.51, 1.91)	7.20 (1.94, 26.76)*	95.43 (24.55, 370.90)*	0.65 (0.26, 1.57)
No	1.00	1.00	1.00	1.00	1.00
Suicidal ideation					
Yes	1.08 (0.30, 3.92)	1.62 (0.61, 4.36)	0.00 (0.000, 0.00)*	0.00 (0.000, 0.00)*	0.96 (0.45, 2.07)
No	1.00	1.00	1.00	1.00	1.00

*AOR* adjusted odds ratio, *CI* confidence interval

*Statistically significant at *p* < 0.05

In addition, males had significantly higher odds of neglecting to brush twice daily (AOR: 1.64; 95%CI 1.04, 2.59); and older adolescents had significantly higher odds of not using condom at the last sexual intercourse (AOR: 1.68; 95%CI 1.35, 2.10) and having multiple sex partners (AOR = 2.83; 95%CI 2.08, 3.85). Adolescents with high and middle socioeconomic status had significantly lower odds of daily consumption of refined carbohydrates in-between-meals (AOR: 0.34; 95%CI 0.24, 0.49 and AOR: 0.50; 95%CI 0.35, 0.70 respectively) and significantly higher odds of having a higher number of risky oral and sexual behaviours (AOR: 2.61; 95%CI 1.87, 3.64 and AOR: 1.84; 95%CI 1.35, 2.51 respectively). In addition, dental service utilisation in the last 12 months was associated with significantly lower odds of risky behaviours—neglecting toothbrushing (AOR: 0.09; 95%CI 0.02, 0.30), none use of condoms at the last sexual intercourse (AOR: 0.00’ 95%CI 0.00, 0.00) and having multiple sex partners (AOR: 0.00’ 95%CI 0.00, 0.00).

## Discussion

This study identified that though psychological distress may increase the risk of having higher numbers of risky oral and sexual behaviours, it also may reduce the risk for daily consumption of refined carbohydrates and having multiple sexual partners. Having depressive symptoms increased the odds for not using condoms and having multiple sex partners while having suicidal ideation reduced the odds for sexual risk behaviours. The study findings highlight the possible relationships between mental, oral and sexual health factors. It provides evidence supporting the call for inter-disciplinary management of adolescents’ health and the needs for professionals to identify risk behaviours that should raise a high index of suspicion for other major health problems.

One of the strengths of the study is the representative data collection method—the use of a household survey—which makes it possible to generalize the study findings to the study population and to other populations with similar epidemiological profile to the study setting. A study limitation however is the possibility of under-reporting sexual risk behaviour due to social desirability bias, especially in a cultural environment like Nigeria where pre-marital sex is frowned at [38]. Also, this is a cross-sectional study and thus, a cause-effect relationship cannot be adduced for the study findings. Also, this study did not collect data on anxiety, thereby limiting the assessment of how this distinct mental health phenomenon may be associated with oral, sexual and reproductive health. Our study findings will need to be corroborated through future studies.

High psychological distress is a major health concern. It leads to withdrawal from physical and social activities for many affected persons. Although having low intake of refined carbohydrates in-between-meals and not having multiple sex partners are good oral and sexual health behaviours, healthcare providers may need to have a high index of suspicion that an adolescent may have psychological distress when these good oral and sexual health behaviours are associated with other factors. Other associated factors that should possibility raise the index of suspicion of psychological distress include high dental anxiety, consumption of alcohol, use of psychoactive substances, and cigarette smoking as the findings in this study indicated.

There is still little understood about the relationship between psychological distress and risky behaviours in adolescents though there are evidence on the associations between the two [39]. There is a reciprocal relationship between psychological distress and the engagement in mood-regulating behaviours [40, 41]. An effective positive mood-regulatory behaviours is exercising [42]. Examples of negative mood-regulatory behaviours are alcohol intake and substance abuse among others [43]. The finding of this study suggests that adolescents in Nigeria in negative mood-regulatory behaviours when psychologically distressed. Prior studies had indicated that adolescents in developed countries have reported high prevalence of multiple health risk behaviours among adolescents [44, 45]. The study conducted in Ghana [46], a country in the same sub-region as Nigeria, indicates that a high prevalence of multiple health risk behaviours among adolescents was also observed in a low-middle income country. This study findings seem to suggest that there may be a clustering of high-risk behaviours associated with psychological distress in adolescents in Nigeria also. These high-risk behaviours are associated with NCDs and risk of mortality, which is a reason why attention need to be paid to the management of psychological distress in Adolescents. Unlike prior studies however, we identified that high dental anxiety is associated with psychological distress. Screening for these psychologically distress and the use of negative mood-regulatory behaviours in the dental clinic in adolescents with high dental anxiety may facilitate the access of adolescents to multiple health behaviour change interventions. More research is needed to identify the model can be best applied to adolescents in Nigeria.

Of interest are the associations found between depressive symptoms, suicidal ideations and sexual health risk behaviours. Some mental health problems like suicidal ideations seem to be associated with less sexual health risk behaviours while others such as depression, are associated with adolescents engaging in risky sexual behaviours. A number of studies had demonstrated an association between depression and high sexual risk behaviour [47, 48]. An explanation is that depression results in negative thoughts and cognitive distortions that can impede healthy decision-making such as safe sexual behaviours and healthy communication about sex such as negotiation to use condom during sex [47]; and allows emotions to influence behaviour [49]. Our study finding provides evidence that the impact of depression on sexual health may be a global phenomenon as studies conducted in North America [50], Asia [51, 52], Latin America [53] and Australia [54] report similar results. Our finding that suicidal ideations reduce the odds for risky sexual behaviour though reported nu Rohde et al. [55], is at variance with findings from a number of prior studies [56, 57] and needs to be explored further.

We also observed some interesting associations. First is that dental service utilisation in the last 12 months was associated with significantly lower odds of neglecting toothbrushing, non-use of condoms at the last sexual intercourse and having multiple sex partners. We think that dental service utilization by adolescents in this population is an indication of low risk-taking behaviour. It may be important to explore if these individuals also have lower odds of consuming alcohol, using psychoactive substances and also smoking cigarettes. A prior study had indicated that the most prevalent risks that adolescents take are sexual risk and substance use which increases the risk for injury and violence [58]. Dangerous risk-taking results from the asynchronous development of the prefrontal cortex and the increase in the white matter in the corpus callosum [58]. This is the first study that indicates that a history of annual dental service utilization may also be an indicator for risk-taking behaviours in adolescents; and a possible discriminant factor for low or high-risk taking adolescents when assessed along with the history of substance use and risky sexual behaviours. This needs to be explored in future studies.

Second is the higher oral, and sexual risk-taking behaviours of adolescents with middle socioeconomic status though they were less likely than adolescents with low socioeconomic status to consume refined carbohydrates in-between-meals daily. Prior studies had highlighted that households with low socioeconomic status tend to have higher rates of consumption of refined carbohydrates because it is cheaper [59, 60]. However, our finding on higher risk-taking behaviour is a reverse of the findings among Slovak adolescents which indicated that adolescents had more risk smoking, alcohol consumption and drug use behaviours than adolescents with higher socio-economic status [61]. It also differed from the finding among adolescents in the Netherlands where there was no significant association between socioeconomic status and smoking, alcohol consumption, drug use [62]. Lee and Seo [63], however, discouraged the use socioeconomic disparity to explain adolescent health behaviours as they observed that stress can partly explain why adolescents engage in multiple risk behaviours irrespective of the social class. Also, the associations between socioeconomic status and health behaviours are not as robust as those in adults [62, 64].

## Conclusion

In conclusion, the associations between mental health and oral, and sexual health behaviours of adolescents appears to be complex and not all associations are in the same direction. The study findings suggest the need for adolescents with high dental anxiety be screened for psychological distress and sexual health risk behaviours. Depression may increase the risk for high sexual risk behaviours. Inter-disciplinary management of adolescents’ health may facilitate prompt diagnosis of mental, sexual and oral health problems in adolescents thereby promptly disrupting the risk for future poor health and wellness for affected individuals.

## Data Availability

All data generated for this study are presented in the manuscript. Patient-level data can however be accessible on reasonable request from the one of the authors, Morenike Oluwatoyin Folayan.

## Abbreviations

AOR: adjusted odds ratio
CI: confidence interval
